# Supplementary material for: Dimensionality‐Controlled Evolution of Charge‐Transfer Energy in Digital Nickelates Superlattices
Source: Adv Sci (Weinh). 2022 May 23;9(21):2105864. doi: 10.1002/advs.202105864 (PMC9313943; doi:10.1002/advs.202105864)
Supplement: Supplementary file 1 — Supporting Information [file ADVS-9-2105864-s001.pdf]

Supporting Information

Dimensionality-controlled evolution of charge-transfer energy in digital nickelates superlattices

Xiangle Lu, Jishan Liu, Nian Zhang, Binping Xie, Shuai Yang, Wanling Liu, Zhicheng Jiang, Zhe Huang, Yichen Yang, Jin Miao, Wei Li, Soohyun Cho, Zhengtai Liu, Zhonghao Liu and Dawei Shen**

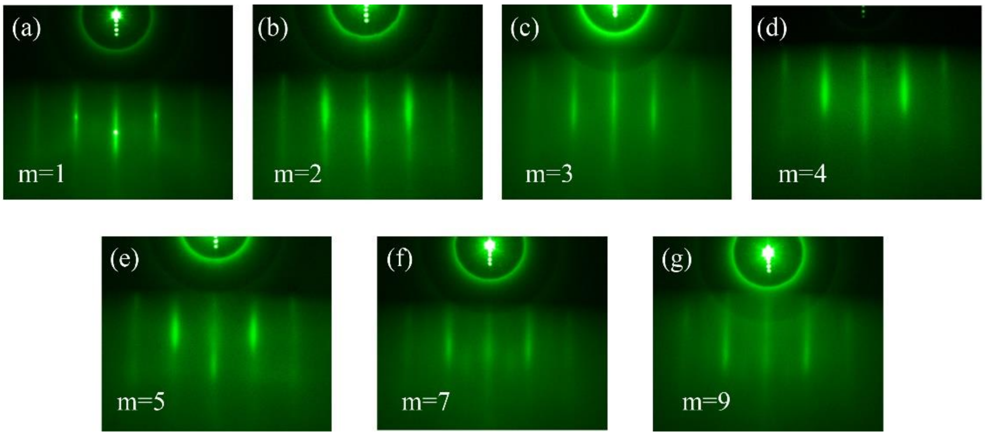

Figure S1. (a)(b)(c)(d)(e)(f)(g) RHEED patterns of superlattices.

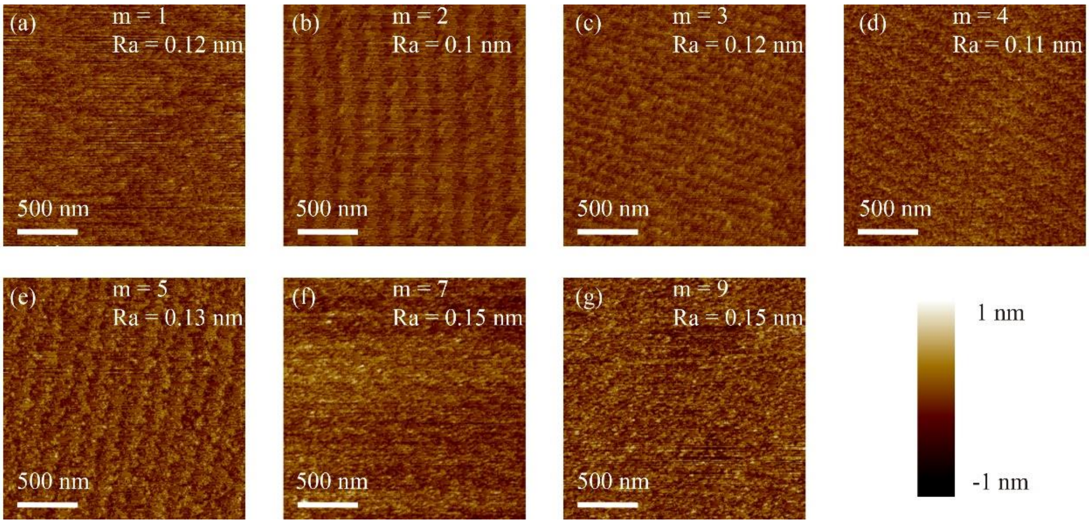

Figure S2. (a)(b)(c)(d)(e)(f)(g) AFM image of superlattices. The lower right corner is a color scale. The average surface roughness Ra of the m = 1, 2, 3, 4, 5, 7, 9 superlattices is 0.12 nm, 0.1 nm, 0.12 nm, 0.11 nm, 0.13 nm, 0.15 nm, 0.15 nm, respectively.

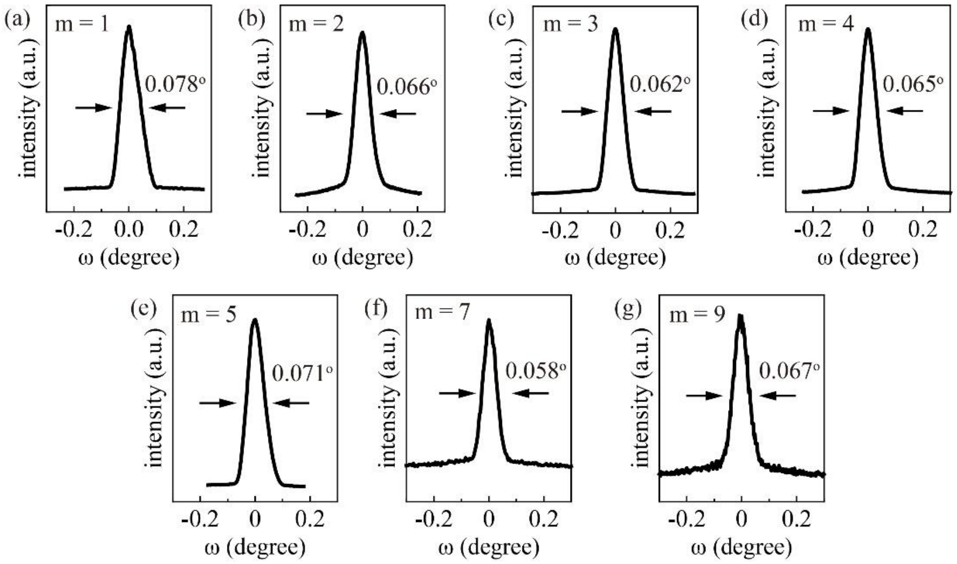

Figure S3. (a)(b)(c)(d)(e)(f)(g) Rocking curve of superlattices with XRD. The full width at half maxima (FWHM) of the m = 1, 2, 3, 4, 5, 7, 9 superlattices is 0.078°, 0.066°, 0.065°, 0.065°, 0.071°, 0.058°, 0.067°, through Gaussian fitting, respectively.

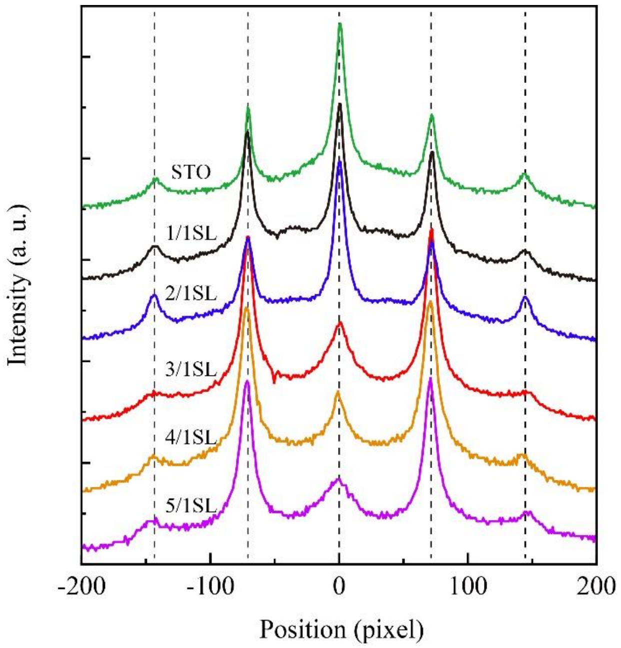

Figure S4. Comparison of RHEED intensity integral curves of $SrTiO_3$ and superlattice films. It clearly shows that the RHEED streak spacing of the SLs match well with those of the $SrTiO_3$ substrates, indicating the coherent growth of the films on $SrTiO_3$ substrates.

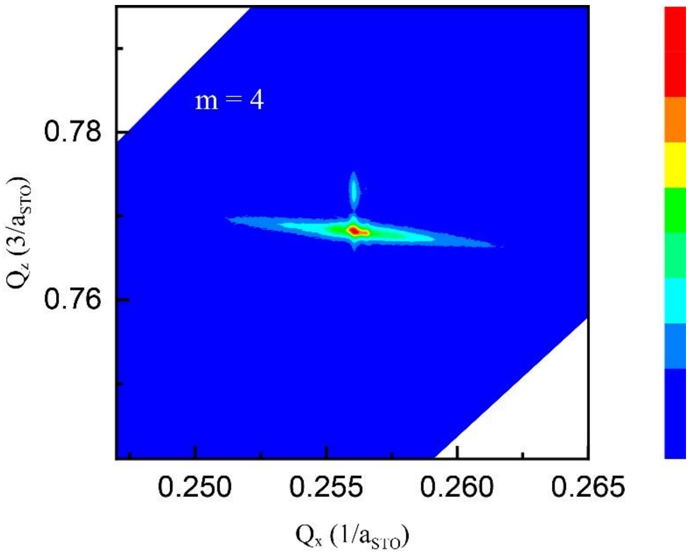

Figure S5. The RSM around (103) reflections for m = 4 SL. Along the horizontal axis, the film is in-plane lattice matched to the $SrTiO_3$ substrate, which confirms that the SL film grown on $SrTiO_3$ is fully strained.

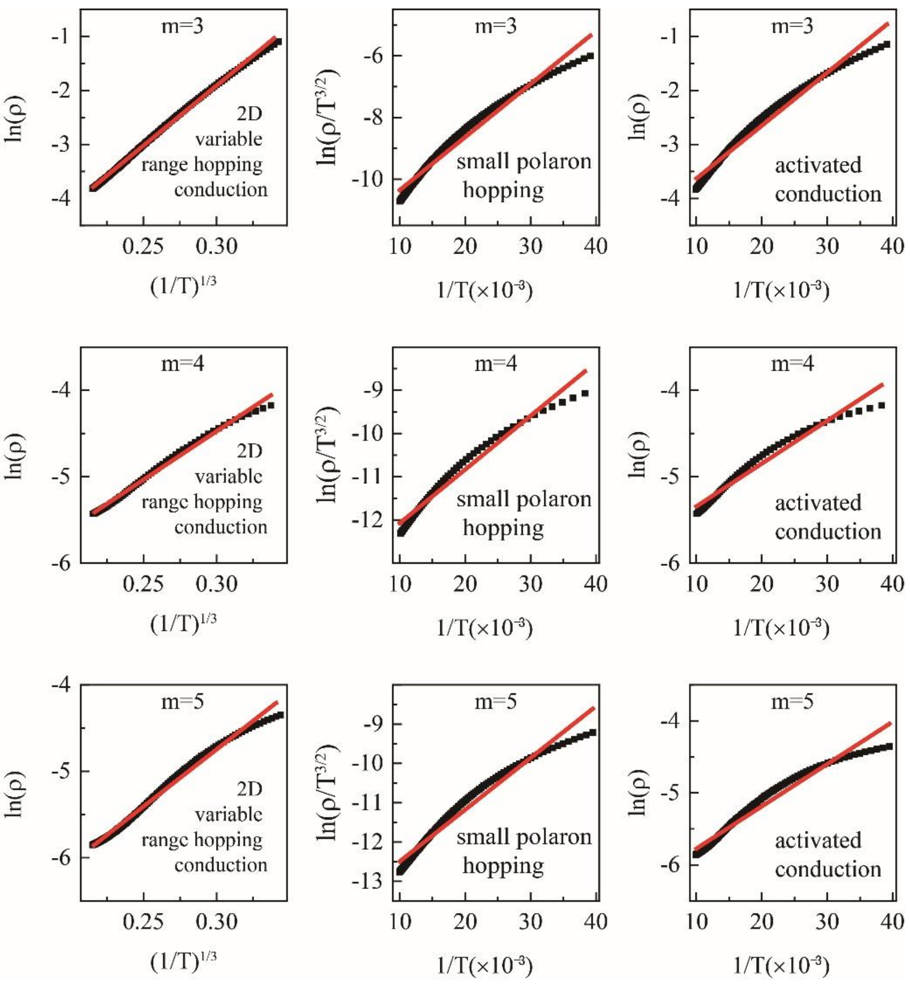

Figure S6. Linear fit (red line) to two-dimensional variable range hopping, small polaron hopping, and activated conduction model for m = 3, 4, 5 SLs, respectively. It is difficult to find a suitable single model to fit well due to the complex resistance-temperature curve in the insulating regions for these samples.

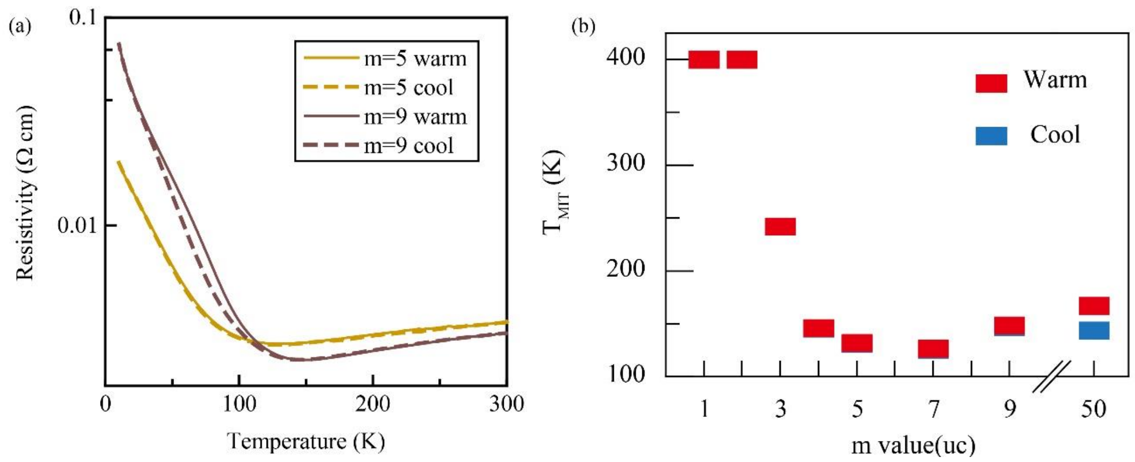

Figure S7. (a) Resistivity versus temperature during warming and cooling for m = 5, 9 SLs. (b) The T_{MIT} of the SLs series obtained by derivation $\mathrm{d}\rho/\mathrm{dT} = 0$ during warming and cooling.

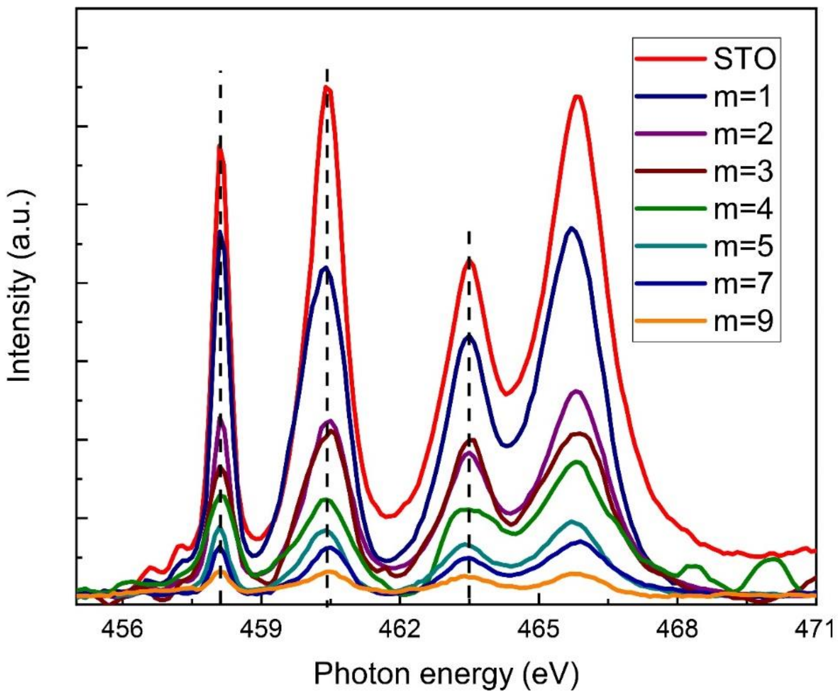

Figure S8. (a) XAS at the Ti *L* edge for the SLs series. No charge transfer happened at the STO/NNO interface for the Ti cation strongly prefers the +4 oxidation state regardless of the thickness of NNO slab.
